# Modulation of Magnetoresistance Polarity in BLG/SL-MoSe_2_ Heterostacks

**DOI:** 10.1186/s11671-020-03365-2

**Published:** 2020-06-22

**Authors:** Muhammad Farooq Khan, Shania Rehman, Malik Abdul Rehman, Muhammad Abdul Basit, Deok-kee Kim, Faisal Ahmed, H. M. Waseem Khalil, Imtisal Akhtar, Seong Chan Jun

**Affiliations:** 1 Department of Electrical Engineering, Sejong University, 209 Neungdong-ro, Gwangjin-gu, Seoul, 05006 South Korea; 2 School of Mechanical Engineering, Yonsei University, 50 Yonsei-ro, Seodaemun-gu, Seoul, 03722 South Korea; 3 Department of Materials Science and Engineering, Institute of Space Technology, Islamabad, 44000 Pakistan; 4 Department of Mechanical Engineering, NUST College of Electrical and Mechanical Engineering, National University of Science and Technology, Islamabad, 44000 Pakistan; 5 Department of Electronics and Nanoengineering, Aalto University, P.O. Box 13500, F1-00076 Aalto, Finland; 6 Department of Electrical Engineering, College of Engineering and Technology, University of Sargodha, Sargodha, Pakistan; 7 Department of Mechanical Engineering, Chung-Ang University, Seoul, South Korea

**Keywords:** Graphene, MoSe_2_, Heterostack, Spin-valve junction, Magnetoresistance, Metals doping

## Abstract

Two-dimensional (2D) layered materials have an atomically thin and flat nature which makes it an ultimate candidate for spintronic devices. The spin-valve junctions (SVJs), composed of 2D materials, have been recognized as unique features of spin transport polarization. However, the magnetotransport properties of SVJs are highly influenced by the type of intervening layer (spacer) inserted between the ferromagnetic materials (FMs). In this situation, the spin filtering effect at the interfaces plays a critical role in the observation of the magnetoresistance (MR) of such magnetic structures, which can be improved by using promising hybrid structure. Here, we report MR of bilayer graphene (BLG), single-layer MoSe_2_ (SL-MoSe_2_), and BLG/SL-MoSe_2_ heterostack SVJs. However, before annealing, BLG and SL-MoSe_2_ SVJs demonstrate positive MR, but after annealing, BLG reverses its polarity while the SL-MoSe_2_ maintains its polarity and demonstrated stable positive spin polarizations at both interfaces due to meager doping effect of ferromagnetic (FM) contacts. Further, Co/BLG/SL-MoSe_2_/NiFe determines positive MR, i.e., ~ 1.71% and ~ 1.86% at *T* = 4 K before and after annealing, respectively. On the contrary, NiFe/BLG/SL-MoSe_2_/Co SVJs showed positive MR before annealing and subsequently reversed its MR sign after annealing due to the proximity-induced effect of metals doping with graphene. The obtained results can be useful to comprehend the origin of polarity and the selection of non-magnetic material (spacer) for magnetotransport properties. Thus, this study established a new paragon for novel spintronic applications.

## Introduction

Transition metal dichalcogenides (TMDs), and graphene are tremendous 2D materials for electronic, photovoltaic, and spintronic devices [1–5]. In spintronics, the SVJ is a promising physical phenomenon and it enables non-volatile data storage with ferromagnetic memory elements acting as a spin polarizer or analyzers. It realized a new-era of magnetic random access memories, magnetic sensors, and basic logic applications as an information vector [6–8]. In recentyears, graphene and two-dimensional transition metal dichalcogenides (2D-TMDs) have found widespread novel spintronic applications [9–16]. They have been used widely to determine high magnetoresistance of 2D materials due to their spin-coherence and high spin-orbit coupling [16, 17]. However, among all TMDs, single-layer MoSe_2_ (SL-MoSe_2_) is less explored in spintronics despite its small spin splitting effect (188 meV) and band gap (1.5 eV) than that for WS_2_ and WSe_2_ in a thin-layer nano-sheet [18, 19]. The integration of SVJs based on 2D materials inherits some issues, such as oxidation resistance, which provokes new development in device fabrication [20–22]. Further, hybrids or heterostructures of 2D layered semiconducting materials and graphene were unexplored in magnetic tunnel junctions. They possibly would have explicit spin properties and complementary information in spin-polarized devices. Several problems of wet transfer in conventional SVJs are those which hammer the adverse oxidation of ferromagnetic metals (FMs) contacts that rely on the quality of interfaces at play to aim the true and high magnetoresistance (MR) values [9, 22, 23]. However, further progress and fabrication of ultimate limit in the size of devices are required to control the oxide barrier, interfaces, substitution of material (spacer), and performance of spin-polarized electrodes.

To overcome these limitations, we exploited 2D materials and their heterostacks to demonstrate proficient, ultra-clean vertical SVJs of three different interlayer junctions between Co and NiFe electrodes. We observed clear spin signals of bilayer graphene (BLG), SL-MoSe_2_, and BLG/SL-MoSe_2_, showing MR up to room temperature. Here, we categorized the spin-valve junctions into two types. In the first type (individual/single materials; either BLG or SL-MoSe_2_) of spin-valve junctions, Co/BLG/NiFe, we investigated the positive and negative spin signals before and after annealing, but in other Co/SL-MoSe_2_/NiFe devices, the spin signal remained positive with a slight improvement in the MR values. Interestingly, in the second type (heterostack; BLG/SL-MoSe_2_) of spin-valve junctions, Co/BLG/SL-MoSe_2_/NiFe, the MR was found to be positive even before and after the annealing process. Moreover, in NiFe/BLG/SL-MoSe_2_/Co devices, a positive MR was observed before annealing, but the spin polarization of the electron reversed with significantly enhanced MR values after annealing.

To explore superior SVJs, decontaminated and residue-free interfaces should be employed for a non-magnetic thin film (spacer) sandwiched between the FM electrodes. An ultra-clean interface of BLG/FMs is achieved by evaporating FMs (without photo- and electron beam-lithography) to circumvent the oxidation process.

## Experimental Methods

### Device Fabrication

The exfoliated BLG is transferred on ~ 2-μm diameter circular hole through a thick SiN window. The suspended BLG film was annealed in a furnace tube in argon and hydrogen gas environment at 350 °C for 4 h to deteriorate the residues from both sides of the suspended part of BLG. Before depositing the FM metals, we irradiated our devices from both sides under a DUV light in a vacuum environment for 15 min to further clean the BLG. Next, Co (~ 20 nm with an evaporation rate = 0.6 Å/s) and Au (~ 5 nm) metals were first deposited on the top side of the suspended graphene, respectively. Subsequently, NiFe (~ 100 nm with an evaporation rate = 0.8 Å/s) and Au (~ 200 nm) were deposited from the bottom side of the sample. Further, to make heterostack BLG was transferred on SL-MoSe_2_ to fabricate a BLG/SL-MoSe_2_ device, which was annealed in a furnace tube in argon (Ar) and hydrogen (H_2_) gas environment at 250 °C for 4 h to deteriorate the residue from both sides of the suspended junction. For SL-MoSe_2_ and BLG/SL-MoSe_2_ devices, Co/Au (35/10 nm) and NiFe/Au (150/200 nm) were deposited on the top and bottom sides, respectively. Then, the devices were annealed in the Ar and H_2_ gas mixture at 250 °C for 15 h to improve the junction quality and its compactness. Details of the hole-drilling process can be seen in Supplementary Information Notes (1-2).

### Device Characterization

A Renishaw Raman micro-spectrometer and a laser wavelength of 514 nm were used to characterize the Raman spectra. Four-probe transport measurements based on vertical spin-valve junctions were performed using an ac lock-in amplifier technique. The driving ac current was fixed at 10 μA for temperature-dependent spin magnetotransport measurements and later increased up to 50 μA to study the effect of current dependence at a constant temperature (*T* = 4 K). The devices were cooled by liquid helium for low-temperature measurements, and the temperature was controlled by Lake Shore 331. The current-voltage measurement was accomplished using a pico-ammeter (Keithley 6485) and a nano-voltmeter (2182A).

## Results and Discussion

### Spin-Valve Junctions of BLG

**Fig. 1:**
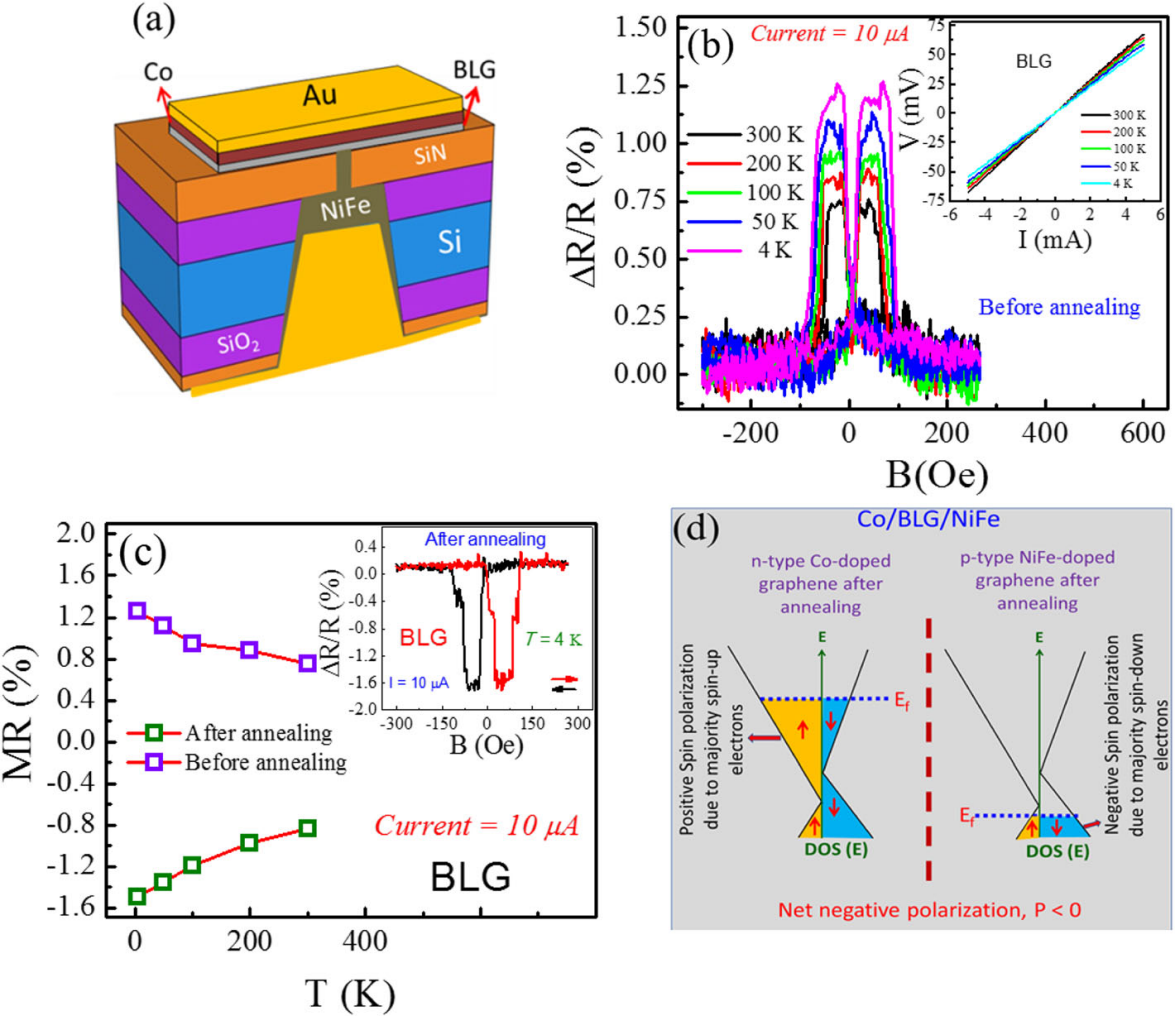
**a** Scheme of device fabrication where ferromagnetic Co and NiFe metals were deposited on the top and bottom, respectively. **b** The change in R vs B traces before annealing at different temperatures (with *I* = 10 μA). (Inset) Current-voltage characteristics of the BLG at different temperatures are linear and indicate an ohmic contact. **c** Temperature-dependent MR values of the BLG before and after annealing at fixed ac current. (Inset) The MR vs B of Co/BLG/NiFe junction after annealing at *T* = 4 K. **d** Schematic drawing of spin-dependent density of states for BLG. Band splitting gives a difference in spin-up and spin-down carriers at *E*_F_. The thick dashed red line in the middle shows decoupling of van der Waals-bonded BLG

In our results, in vertical SVJ, BLG is sandwiched between Co and NiFe electrodes; its schematic is shown in Fig. 1a. From Figure S1a, the Raman spectrum of suspended region confirms BLG as the G, and 2D peaks were found near ~ 1585.5 and ~ 2710 cm^−1^, respectively, which is consistent with a previous report [24]. In addition, after FM depositions, the scanning electron microscopy (SEM) image of the top side is shown in Figure S1b. Thereafter, temperature-dependent *I-V* characteristics were obtained, as shown in Fig. 1b (inset) where valuable information about conducting behavior of the SVJ was demonstrated. Figure 1b (inset) shows the linear curves for FM/BLG/FM, an indication of an ohmic contact, which is consistent with a previous report [25]. The change in R vs B (in-plane) at different temperatures was observed as shown in Fig. 1b. The two electrodes were magnetically separated and switched independently at room temperature, where MR is defined as MR (%) = [(*R*_AP_ − *R*_P_)/*R*_P_] × 100 (%). Here, *R*_AP_ corresponds to the resistance when the magnetizations of the FM layers align in an anti-parallel configuration, and *R*_P_ is the resistance when the magnetizations of the FM layers are aligned parallel. Since, before annealing, we measured the devices and found positive magnetoresistance for BLG SVJ, representing low- and high-resistance states due to parallel and anti-parallel alignment of magnetizations of the FM materials, respectively. Figure 1b shows the MR traces at different temperatures by fixing constant current value (*I* = 10 μA). It was found that before annealing the MR values of BLG increased monotonically from ~ 0.75, ~ 0.88, ~ 0.95, ~ 1.12, and ~ 1.26% at *T* = 300, 200, 100, 50, and 4 K, respectively, as shown in Fig. 1c. However, these results are consistent and comparatively better than previous reports [26–28]. A higher magnetoresistance was observed at a low temperature, which is typical behavior of magnetic tunnel junctions (MTJs) attributed to the excitation of spin waves in FM materials [29]. Therefore, after annealing, the BLG SVJ changes its sign due to the doping effect of Co and NiFe on both the top and bottom sides of BLG as shown in Fig. 1c (inset). Importantly, after annealing, the MR is increased to ~ − 0.84, ~ − 0.98, ~ − 1.19, ~ − 1.35, and ~ − 1.49% at *T* = 300, 200, 100, 50, and 4 K, respectively, as shown in Fig. 1c. Thus, the spin polarization is reversed and suggests a negative MR, which is attributed to charge transfer and proximity-induced band splitting in BLG as shown in Fig. 1d [28].

Due to annealing the junction becomes compact, and the distance between the layers and junction resistance is reduced (Figure S3c); otherwise, before annealing, there could be a few angstrom (Å) gaps that act as insulators, hinder the doping mechanism, and circumvent the proximity-induced band splitting effect as reported in a previous report [28]. In addition, at Fermi level, spin-up electrons are in a majority in n-doped graphene, while spin-down electrons are the majority in p-doped graphene which generates a negative MR. Moreover, to confirm the doping effect of Co and NiFe, we fabricated the field-effect transistors of pristine BLG, Co-doped BLG, and NiFe-doped BLG as shown in Figure S3(a,b). We have used Ni_89_Fe_11_, therefore, Ni easily can dope p-type as reported previously [30, 31]. The Dirac measurements show that the charge neutrality point (CNP) of pristine BLG lies near + 4 V. After doping of BLG with Co and NiFe, the CNP shifted to + 17 and − 11 V, respectively, which endorse the modulation of Fermi level of BLG, as shown in Figure S3b.

### Spin-Valve Junction of SL-MoSe_2_

**Fig. 2:**
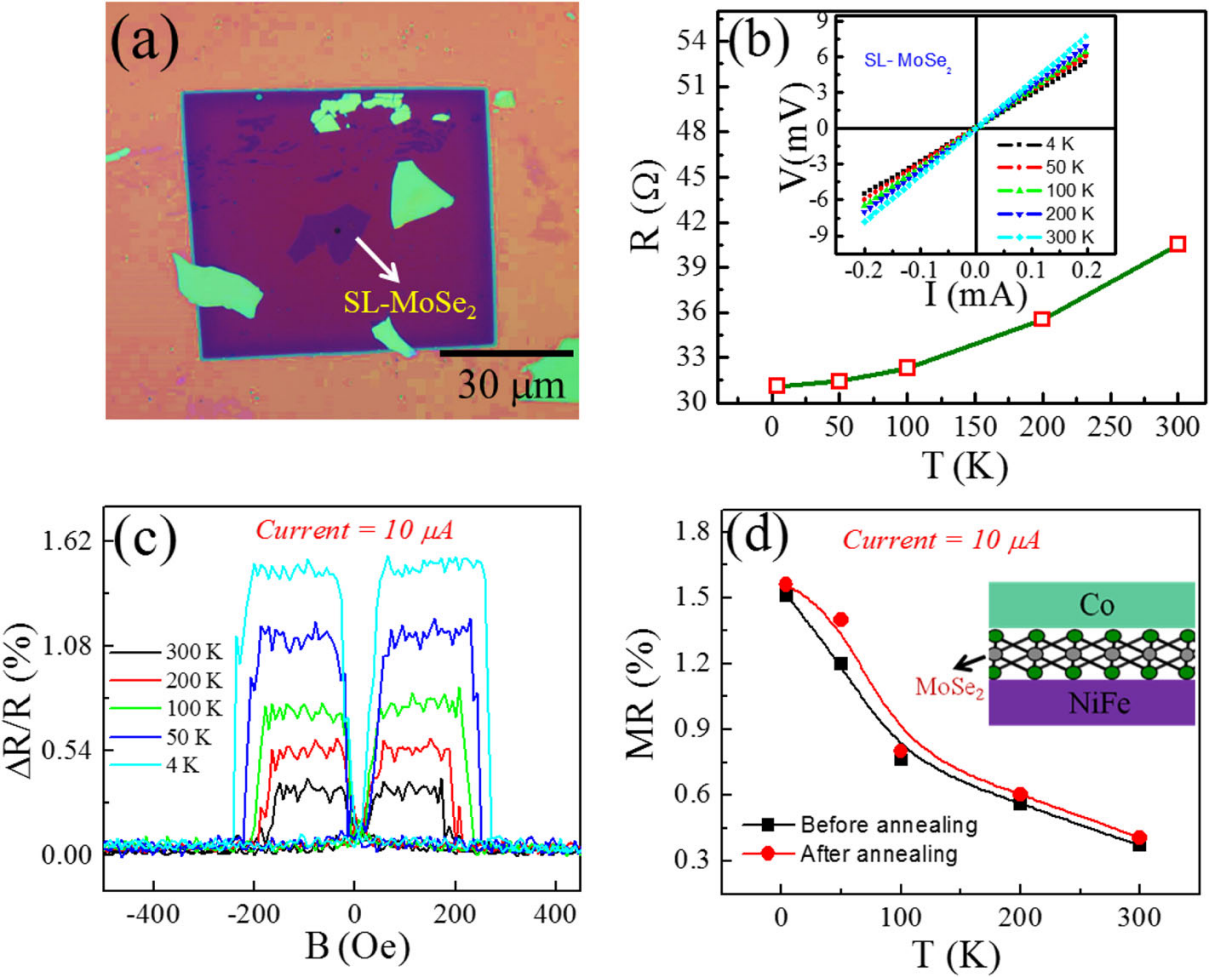
**a** Optical image of SL-MoSe_2_ flake on top of the hole. **b** Junction resistance of SL-MoSe_2_ at different temperatures. (Inset) Temperature-dependent *I*-*V* curves of vertical Co/SL-MoSe_2_/NiFe SVJ demonstrates a metallic junction. **c** The variation of R vs B at *T* = 300, 200, 100, 50, and 4 K before annealing. **d** The temperature-dependent MR ratio of Co/SL-MoSe_2_/NiFe before and after annealing at fixed current. (Inset) The schematic illustration of the device with SL-MoSe_2_

Moreover, the optical image of SL-MoSe_2_ transferred on the SiN membrane hole is depicted in Fig. 2a. The height of the exfoliated MoSe_2_ flake, measured by atomic force microscopy (AFM), and the height profile suggest ~ 0.7 nm thick as shown in Figure S2a-b. In single-layer exfoliated MoSe_2_, the A_1g_ (out-of-plane) Raman mode softens to ~ 240.6 cm^−1^ and the E^1^_2g_ (in-plane) mode stiffens to ~ 286.4 cm^−1^, as shown in Figure S2c, which is consistent with the previous reports [32]. The junction resistance of Co/SL-MoSe_2_/NiFe spin-valve junction is shown in Fig. 2b, which decreased with decreasing temperature. Further, in the linear *I-V* curves at different temperatures, inset of Fig. 2b also reveals an ohmic contact between the SL-MoSe_2_ and the FM electrodes. The linear *I-V* characteristics suggest that the monolayer MoSe_2_ acts as a conducting thin film rather than a tunnel barrier between the electrodes. In Fig. 2c, the MR loops of Co/SL-MoSe_2_/NiFe have been shown at different temperatures by keeping a constant current (*I* = 10 μA), which generates a positive spin signal. The scheme of SL-MoSe_2_ SVJ is shown inset in Fig. 2d. The temperature-dependent MR values for the Co/SL-MoSe_2_/NiFe junction are shown in Fig. 2d, where it is observed that MR reduces as the temperature increases.

In this junction, the MR magnitudes at *I* = 10 μA are determined to be ~ 0.37, ~ 0.56, ~ 0.76, ~ 1.2, and ~ 1.51% at *T* = 300, 200, 100, 50, and 4 K, respectively. Additionally, at a fixed ac current, the MR values of Co/SL-MoSe_2_/NiFe junction enhanced slightly after annealing the devices and reached up to ~ 0.41, ~ 0.6, ~ 0.79, ~ 1.4, and ~ 1.56% at *T* = 300, 200, 100, 50, and 4 K, respectively, as shown in Fig. 2d. Thus, the enhancement of MR could be ascribed to improvement of junction quality, as indicated in Figure S3c, where the junction resistance of all the devices reduced significantly after annealing. Importantly, the polarity of these SL-MoSe_2_ junctions remained the same, since Co and NiFe did not dope SL-MoSe_2_ enough to shift its Fermi level from the conduction band to valence band or vice versa. That is why MoSe_2_ demonstrated stable positive spin polarization at both interfaces.

### Spin-Valve Junction of BLG/SL-MoSe_2_ Heterostack

**Fig. 3:**
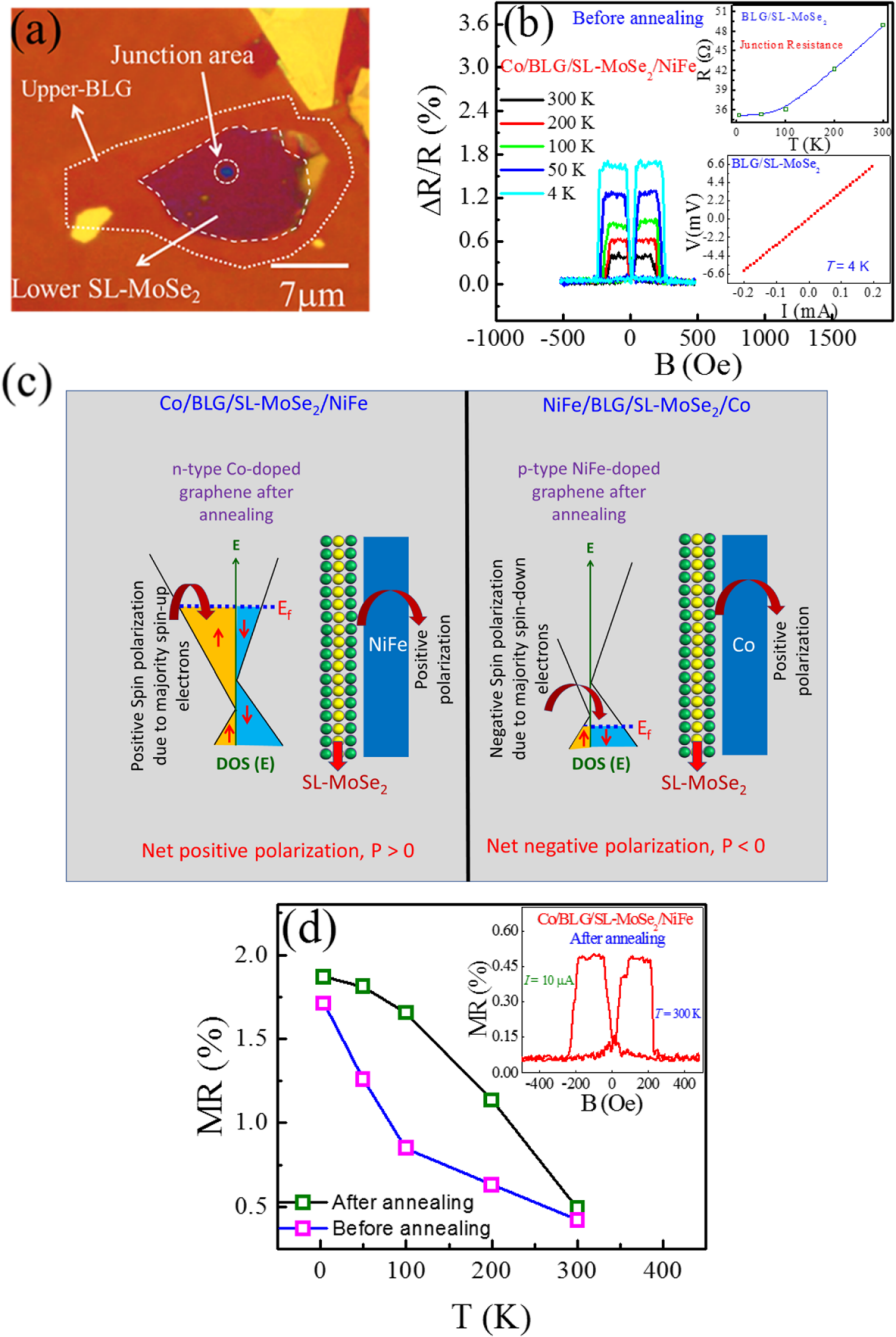
**a** Optical microscopic image of BLG/SL-MoSe_2_ on a hole. **b** The temperature-dependent MR loops of Co/BLG/SL-MoSe_2_/NiFe junction at fixed current (*I* = 10 μΑ). (Top-inset) The temperature-dependent junction resistance of Co/BLG/SL-MoSe_2_/NiFe. (Bottom-inset) The linear *I-V* curve of Co/BLG/SL-MoSe_2_/NiFe device at *T* = 4 K. **c** Schematic drawing of spin-dependent density of states for BLG and SL-MoSe_2_ heterostacks. After annealing the devices, the Fermi levels of BLG adjacent to the Co or NiFe are shifted due to n-type or p-type doping. **d** Before and after annealing, the MR magnitudes as a function of temperature for the structure of Co/BLG/SL-MoSe_2_/NiFe. (Inset) After annealing, the temperature-dependent MR loop of the Co/BLG/SL-MoSe_2_/NiFe junction at a fixed current, *I* = 10 μΑ

The heterostack of atomically thin 2D materials was explored owing to its distinct spin-polarized transport properties. Further, the optical image of BLG/SL-MoSe_2_ heterostack on the SiN hole is shown in Fig. 3a. The temperature-dependent junction resistance is shown in Fig. 3b (top-inset), wherein the resistance decreases with decreasing temperature, which indicates a metallic junction. For further confirmation of the metallic behavior, we investigated the four-probe geometry *I-V* characteristic at *T* = 4 K shown in Fig. 3b (bottom-inset). The Co/BLG/SL-MoSe_2_/NiFe junction exhibits a linear *I-V* curve owing to an ohmic contact. Before annealing, Fig. 3b shows the positive MR traces, which demonstrate the positive spin polarization in Co/BLG/SL-MoSe_2_/NiFe. However, after annealing, the MR sign remained positive (Fig. 3d, inset) and the values increased from ~ 0.42, ~ 0.63, ~ 0.85, ~ 1.26, and ~ 1.71% (Fig. 3d; before annealing) to ~ 0.49, ~ 1.13, ~ 1.65, ~ 1.81, and ~ 1.86% (Fig. 3d; after annealing) at *T* = 300, 200, 100, 50, and 4 K, respectively, as shown in Fig. 3d. High MR values at low temperatures are typical behavior of the spin-valve junctions [33, 34]. The positive MR in the Co/BLG/SL-MoSe_2_/NiFe devices is attributed to similar positive spin polarizations of both interfaces: Co/BLG and SL-MoSe_2_/NiFe. In our findings, we elucidate the positive spin polarization in SL-MoSe_2_ (Fig. 2c), while in Co/BLG/NiFe spin-valve junction, the Co/BLG interface also gives rise to the positive spin polarization. Thus, the net polarization of Co/BLG/SL-MoSe_2_/NiFe spin-valve junctions is positive which is explained schematically in Fig. 3c.

**Fig. 4:**
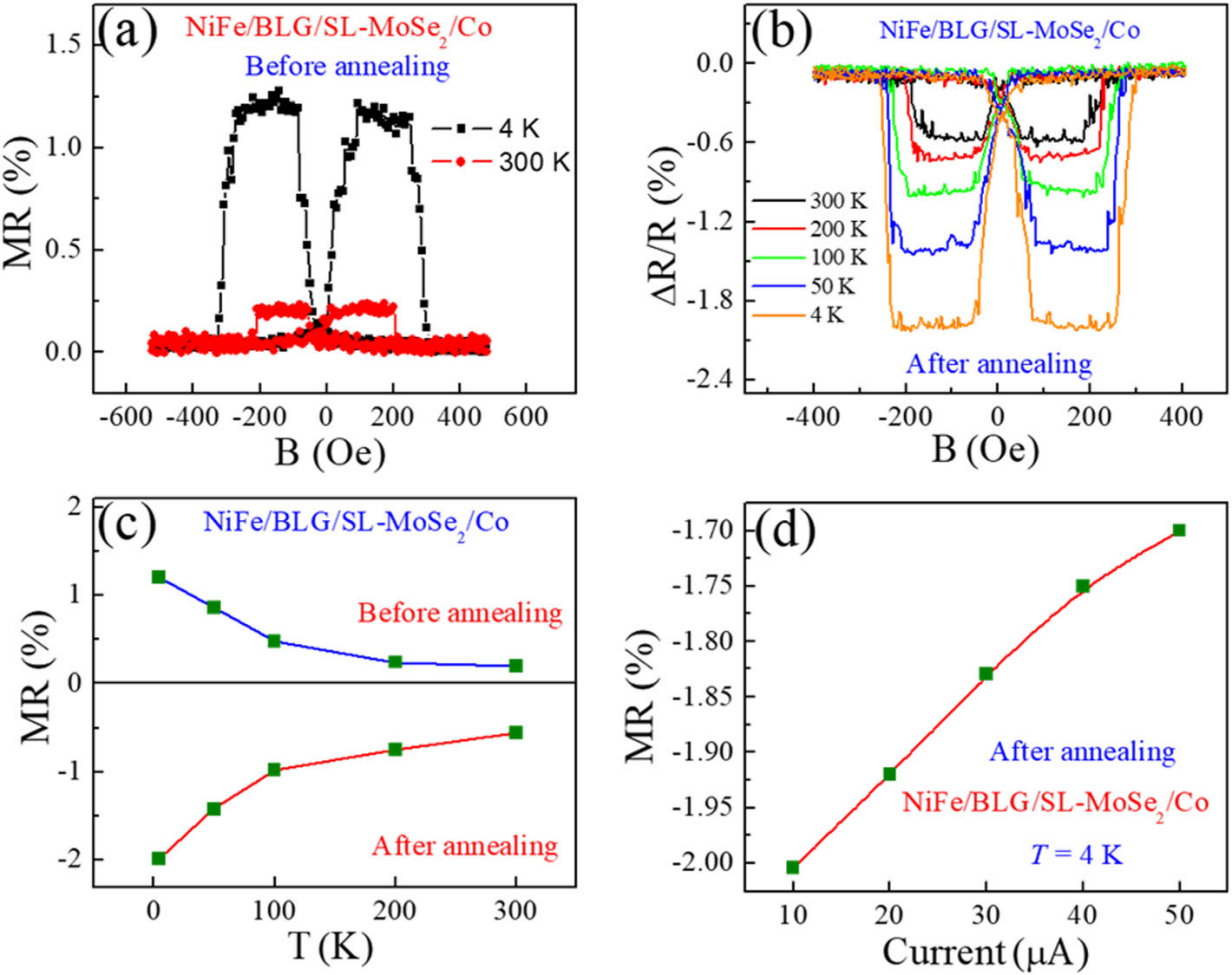
**a** Before annealing, the MR traces as a function of the magnetic field at *T* = 300, 4 K and *I* = 10 μA. **b** After annealing, the MR traces vs magnetic field, B, at different temperatures. **c** Before and after annealing, the MR values at *T* = 300, 200, 100, 50, and 4 K. **d** The MR magnitudes of NiFe/BLG/SL-MoSe_2_/Co at different current values

Moreover, to elucidate the role of Co and NiFe doping with BLG, we fabricated another set of heterostack devices, NiFe/BLG/MoSe_2_/Co. Before annealing, we measured the MR loops that described positive magnetoresistance, as shown in Fig. 4a. Importantly, after annealing, the polarity of NiFe/BLG/MoSe_2_/Co junction reversed, as shown in Fig. 4b. The negative polarization is attributed to hole-doping on the NiFe/BLG interface and proximity-induced band splitting in BLG, which induces the majority of spin-down electrons [28]. The temperature-dependent MR values of the NiFe/BLG/MoSe_2_/Co SVJs were calculated (~ 0.12, ~ 0.24, ~ 0.48, ~ 0.86, and ~ 1.2% at *T* = 300, 200, 100, 50, and 4 K, before annealing and ~ -0.56, ~ -0.75, ~ -0.98, ~ -1.42, and ~ -1.99% at *T* = 300, 200, 100, 50, and 4 K, after annealing) as shown in Fig. 4c. It is notable that after annealing, the MR values increased due to decreased resistance, gaps between layers, and improved doping phenomenon in BLG by NiFe. Further, before and after annealing the net polarization of NiFe/BLG/SL-MoSe_2_/Co SVJ is positive and negative, respectively which is illustrated schematically in Fig. 3c. In addition, after annealing the current-dependent MR, ratios of the NiFe/BLG/MoSe_2_/Co SVJ were calculated as shown in Fig. 4d. Therefore, it was found that with increasing ac current from *I* = 10 μA to *I* = 50 μA, the MR value decreased from ~ − 2.0 to ~ − 1.71%. This reduction of MR is conventional and due to the spin excitations localized at the interfaces and the local trap states in non-magnetic spacer [13, 15, 35, 36]. At this end, we plotted a graph which presents the MR (%) values of our all types of devices throughout this project and revealed a consistent and repeatable trend as shown in Figure S4.

However, doping due to the FM contacts [37] and band splitting due to the proximity effect create a difference in the population of the spin-up and spin-down electrons in graphene [38, 39]. Upon annealing, the conformation and improved contact between the FM contacts and adjacent bilayer graphene provide an effective decoupling of graphene layers within a van der Waals-bonded few-layer crystal, as reported in the twisted graphene bilayers making two electronically decoupled thinner graphenes [40]. Afterward, these two distinctly doped and proximitized graphene layers become spin-polarized electrodes, which decide the polarity of magnetoresistance.

Basically, Co and NiFe FMs have n- and p-type doping in BLG, respectively. In combination with Co/BLG, the Fermi level of BLG is moved to the conduction band due to n-doping. When the Fermi level of BLG lies in the conduction band, the density or population of the spin-up electrons increases as compared to the spin-down electrons due to the proximity-induced band splitting of graphene, ultimately presenting a positive spin polarization. On the other hand, in NiFe/BLG stack, the Fermi level of BLG shifted to the valence band and proximity-induced band splitting encourages the density of the spin-down electron, which finally demonstrates a negative spin polarization. Notably, in our experiments, the proximity-induced effect in BLG becomes prominent only when the devices are annealed after metallization of the FMs as observed similarly in ref. [28]. Initially, we were interested about the Fermi-level of SL-MoSe_2_ that may possibly move due to proximitized contact of Co or NiFe after the annealing process. But surprisingly, it remained consistent due to the meager doping effect on MoSe_2_. It demonstrated stable positive spin polarizations at SL-MoSe_2_/NiFe and SL-MoSe_2_/Co interfaces due to which we can easily modulate the sign of MR by selection of either NiFe or Co with BLG in Co/BLG/SL-MoSe_2_/NiFe or NiFe/BLG/SL-MoSe_2_/Co junctions. In addition, we have found that in ref. [28], a maximum of 1% MR is observed after annealing in BLG spin-valve junction. On the other hand, in our work after annealing, we have found MR ~ 1.86% (86% larger than that of ref. [28]) in Co/BLG/SL-MoSe_2_/NiFe and ~ 1.99% (99% larger than that of ref. [28]) in NiFe/BLG/SL-MoSe_2_/Co devices. Since, we concluded that the manifestation of BLG/SL-MoSe_2_ junction provides large MR values as compared to only BLG or SL-MoSe_2_, thus, the basic functionality of device fabrication may contribute to opening a new avenue for logic and memory spintronic applications in the future.

## Conclusions

In summary, we revealed decontaminated SVJs of Co/BLG/NiFe, Co/SL-MoSe_2_/NiFe, Co/BLG/SL-MoSe_2_/NiFe, and NiFe/BLG/SL-MoSe_2_/Co. The current-voltage characteristic of all SVJs demonstrated a linear relation, which confirmed the metallic junction and behaves like conducting film. We examined the positive and negative MR signals in Co/BLG/NiFe before and after annealing, respectively. Since after annealing, the proximity-induced effect reverses the polarity of BLG SVJs. Although, in the Co/SL-MoSe_2_/NiFe, the MR values have improved faintly, but unlike BLG, its polarity remained the same (positive) before and after annealing because SL-MoSe_2_ has a negligible doping effect from FMs. Moreover, like SL-MoSe_2_ the heterostack SVJs of Co/BLG/SL-MoSe_2_/NiFe showed a positive polarity before and after the annealing process, but its MR values are significantly enhanced after annealing. Additionally, NiFe/BLG/MoSe_2_/Co SVJs demonstrated a positive MR before annealing, but after annealing, the polarity is reversed due to proximity-induced band splitting of BLG coupled with NiFe with improved MR values. Moreover, we observed the current-dependent MR magnitudes which decrease at large current values and are attributed to the contribution of interfacial states at high biases. Hence, compared to BLG and SL-MoSe_2_, the BLG/SL-MoSe_2_ heterostack reveals higher MR and spin polarizations, thereby proposing better spin filtering phenomenon at the interfaces. Subsequently, in BLG/SL-MoSe_2_ devices, the polarity is not only reversed but also it demonstrates the efficient spin filtering mechanism at FM interfaces. These investigations on 2D semiconductor materials and their heterostacks may explore valuable complementary information in spintronic logic devices.

## Supplementary information

**Additional file 1: Supplementary Note 1.** Fabrication of hole through wafers. **Supplementary Note 2.** Schematic illustration of device fabrication for hole. **Figure S1.** Characterizations of suspended graphene structure. (a) The Raman spectrum of bilayer suspended graphene. The small D peak is observed which attributed to strain effect and is normal in suspended graphene. (b) After FMs depositions the Scanning electron microscopy (SEM) image of final device from top side. **Figure S2.** (a) The AFM image of single layer MoSe_2_ flake is taken on substrate. (b) The height profile corresponding to thin MoSe_2_ shows single layer as the thickness of our flake is very close to reported value (~0.77 nm). (c) The Raman spectrum of single layer MoSe_2_ on supported region. The A_1g_ and E^1^_2g_ peaks are observed around ~240.6 and 286.4 cm-1 which is also sign of single layer MoSe_2_. **Figure S3.** (a) Schematic drawing of graphene FETs with Co and NiFe doping. (b) The resistivity *vs* back gate, Dirac measurements. (c) The RA of the junction devices before and after annealing. The resistance of all devices is reduced after annealing. **Figure S4.** The MR (%) values at different temperature for all type of devices after annealing by keeping current I = 10 μA.

## Data Availability

The authors have no data to share since all data is already shown in the submitted manuscript.

## Abbreviations

TMDs: Transition metal dichalcogenides
2D: Two-dimensional
MR: Magnetoresistance
BLG: Bilayer graphene
SL-MoSe_2_: Single-layer MoSe_2_
CNP: Charge neutrality point
AFM: Atomic force microscopy

## References

[1] Dayen J-F, Ray SJ, Karis O, Vera-Marun IJ, Kamalakar MV (2020). Two-dimensional van der Waals spinterfaces and magnetic-interfaces. Appl Phys Rev.

[2] Wang Z, Sapkota D, Taniguchi T, Watanabe K, Mandrus D, Morpurgo AF (2018). Tunneling spin valves based on Fe3GeTe2/hBN/Fe3GeTe2 van der Waals heterostructures. Nano Lett.

[3] Khan MF, Nazir G, Lermolenko VM, Eom J (2016). Electrical and photo-electrical properties of MoS2 nanosheets with and without an Al2O3 capping layer under various environmental conditions. Sci Technol Adv Mater.

[4] Rehman MA, Roy SB, Gwak D, Akhtar I, Nasir N, Kumar S, Khan MF, Heo K, Chun S-H, Seo Y (2020) Solar cell based on vertical graphene nano hills directly grown on silicon. Carbon.

[5] Khan MF, Rehman S, Akhtar I, Aftab S, Ajmal HMS, Khan W, Kim D-K, Eom J (2019). High mobility ReSe2 field effect transistors: Schottky-barrier-height-dependent photoresponsivity and broadband light detection with Co decoration. 2D Materials.

[6] Chappert C, Fert A, Van Dau FN (2007). The emergence of spin electronics in data storage. Nat Mater.

[7] Childress JR, Fontana RE (2005). Magnetic recording read head sensor technology. Cr Phys.

[8] Dery H, Dalal P, Cywinski L, Sham LJ (2007). Spin-based logic in semiconductors for reconfigurable large-scale circuits. Nature.

[9] Wang WY, Narayan A, Tang L, Dolui K, Liu YW, Yuan X, Jin YB, Wu YZ, Rungger I, Sanvito S, Xiu FX (2015). Spin-valve effect in NiFe/MoS2/NiFe junctions. Nano Lett.

[10] Dankert A, Pashaei P, Kamalakar MV, Gaur APS, Sahoo S, Rungger I, Narayan A, Dolui K, Hoque MA, Patel RS, de Jong MP, Katiyar RS, Sanvito S, Dash SP (2017). Spin-polarized tunneling through chemical vapor peposited multilayer molybdenum disulfide. ACS Nano.

[11] Khan MF, Kim H, Nazir G, Jung S, Eom J (2018). Layer dependent magnetoresistance of vertical MoS 2 magnetic tunnel junctions. Nanoscale.

[12] Zhao KK, Xing YH, Han J, Feng JF, Shi WH, Zhang BS, Zeng ZM (2017). Magnetic transport property of NiFe/WSe2/NiFe spin valve structure. J Magn Magn Mater.

[13] Iqbal MZ, Iqbal MW, Siddique S, Khan MF, Ramay SM (2016) Room temperature spin valve effect in NiFe/WS2/Co junctions. Sci Rep Uk 6 10.1038/srep21038 PMC4751526 26868638

[14] Avsar A, Tan JY, Kurpas M, Gmitra M, Watanabe K, Taniguchi T, Fabian J, Ozyilmaz B (2017). Gate-tunable black phosphorus spin valve with nanosecond spin lifetimes. Nat Phys.

[15] Xu LL, Feng JF, Zhao KK, Lv WM, Han XF, Liu ZY, Xu XH, Huang H, Zeng ZM (2017) Magnetoresistance effect in NiFe/BP/NiFe vertical spin valve devices. Adv Cond Matter Phys Artn 9042823. 10.1155/2017/9042823

[16] Tombros N, Jozsa C, Popinciuc M, Jonkman HT, van Wees BJ (2007). Electronic spin transport and spin precession in single graphene layers at room temperature. Nature.

[17] Gong ZR, Liu GB, Yu HY, Xiao D, Cui XD, Xu XD, Yao W (2013) Magnetoelectric effects and valley-controlled spin quantum gates in transition metal dichalcogenide bilayers. Nat Commun:4 10.1038/ncomms3053 23784147

[18] Tongay S, Zhou J, Ataca C, Lo K, Matthews TS, Li JB, Grossman JC, Wu JQ (2012). Thermally driven crossover from indirect toward direct bandgap in 2D semiconductors: MoSe2 versus MoS2. Nano Lett.

[19] Reyes-Retana J, Cervantes-Sodi F (2016). Spin-orbital effects in metal-dichalcogenide semiconducting monolayers. Sci Rep.

[20] Dlubak B, Martin M-B, Weatherup RS, Yang H, Deranlot C, Blume R, Schloegl R, Fert A, Anane A, Hofmann S (2012). Graphene-passivated nickel as an oxidation-resistant electrode for spintronics. ACS Nano.

[21] Cobas E, Friedman AL, van’t Erve OM, Robinson JT, Jonker BT (2012). Graphene as a tunnel barrier: graphene-based magnetic tunnel junctions. Nano Lett.

[22] Piquemal-Banci M, Galceran R, Martin M-B, Godel F, Anane A, Petroff F, Dlubak B, Seneor P (2017). 2D-MTJs: introducing 2D materials in magnetic tunnel junctions. J Phys D Appl Phys.

[23] Dankert A, Kamalakar MV, Wajid A, Patel RS, Dash SP (2015). Tunnel magnetoresistance with atomically thin two-dimensional hexagonal boron nitride barriers. Nano Res.

[24] Annamalai M, Mathew S, Jamali M, Zhan D, Palaniapan M (2013). Effects of annealing on the ripple texture and mechanical properties of suspended bilayer graphene. J Phys D Appl Phys.

[25] Entani S, Seki T, Sakuraba Y, Yamamoto T, Takahashi S, Naramoto H, Takanashi K, Sakai S (2016) Magnetoresistance effect in Fe20Ni80/graphene/Fe20Ni80 vertical spin valves. Appl Phys Lett 109(8)

[26] Li W, Xue L, Abruna HD, Ralph DC (2014) Magnetic tunnel junctions with single-layer-graphene tunnel barriers. Phys Rev B 89(18)

[27] Meng J, Chen JJ, Yan Y, Yu DP, Liao ZM (2013). Vertical graphene spin valve with Ohmic contacts. Nanoscale.

[28] Asshoff P, Sambricio J, Rooney A, Slizovskiy S, Mishchenko A, Rakowski A, Hill E, Geim A, Haigh S, Fal’Ko V (2017). Magnetoresistance of vertical Co-graphene-NiFe junctions controlled by charge transfer and proximity-induced spin splitting in graphene. 2D Materials.

[29] Shang CH, Nowak J, Jansen R, Moodera JS (1998). Temperature dependence of magnetoresistance and surface magnetization in ferromagnetic tunnel, junctions. Phys Rev B.

[30] Lee SI, Song W, Kim Y, Song I, Jung DS, Jung MW, Cha M-J, Park SE, An K-S, Park C-Y (2013). P-type doping of graphene films by hybridization with nickel nanoparticles. Jpn J Appl Phys.

[31] Leong WS, Gong H, Thong JT (2014). Low-contact-resistance graphene devices with nickel-etched-graphene contacts. ACS Nano.

[32] Xia J, Huang X, Liu LZ, Wang M, Wang L, Huang B, Zhu DD, Li JJ, Gu CZ, Meng XM (2014). CVD synthesis of large-area, highly crystalline MoSe2 atomic layers on diverse substrates and application to photodetectors. Nanoscale.

[33] Åkerman J, Roshchin I, Slaughter J, Dave R, Schuller I (2003). Origin of temperature dependence in tunneling magnetoresistance. EPL (Europhysics Letters).

[34] Jansen R, Moodera J (2000). Magnetoresistance in doped magnetic tunnel junctions: effect of spin scattering and impurity-assisted transport. Phys Rev B.

[35] Zhang S, Levy PM, Marley AC, Parkin SSP (1997). Quenching of magnetoresistance by hot electrons in magnetic tunnel junctions. Phys Rev Lett.

[36] Tsymbal EY, Mryasov ON, LeClair PR (2003). Spin-dependent tunnelling in magnetic tunnel junctions. J Phys Condens Mat.

[37] Khomyakov P, Giovannetti G, Rusu P, Karpan V, van den Brink J, Kelly PJ (2008) Doping graphene with metal contacts. 10.1103/PhysRevLett.101.026803 18764212

[38] Wang Z, Tang C, Sachs R, Barlas Y, Shi J (2015). Proximity-induced ferromagnetism in graphene revealed by the anomalous Hall effect. Phys Rev Lett.

[39] Sakai S, Majumdar S, Popov ZI, Avramov PV, Entani S, Hasegawa Y, Yamada Y, Huhtinen H, Naramoto H, Sorokin PB (2016). Proximity-induced spin polarization of graphene in contact with half-metallic manganite. ACS Nano.

[40] San-Jose P, Gorbachev R, Geim A, Novoselov K, Guinea F (2014). Stacking boundaries and transport in bilayer graphene. Nano Lett.

