# Supplementary material for: Modulation of Magnetoresistance Polarity in BLG/SL-MoSe2 Heterostacks
Source: Nanoscale Res Lett. 2020 Jun 22;15:136. doi: 10.1186/s11671-020-03365-2 (PMC7310050; doi:10.1186/s11671-020-03365-2)
Supplement: Supplementary file 1 — Additional file 1: Supplementary Note 1. Fabrication of hole through wafers. Supplementary Note 2. Schematic illustration of device fabrication for hole. Figure S1. Characterizations of suspended graphene structure. (a) The Raman spectrum of bilayer suspended graphene. The small D peak is observed which attributed to strain effect and is normal in suspended graphene. (b) After FMs depositions the Scanning electron microscopy (SEM) image of final device from top side. Figure S2. (a) The AFM image of single layer MoSe2 flake is taken on substrate. (b) The height profile corresponding to thin MoSe2 shows single layer as the thickness of our flake is very close to reported value (~0.77 nm). (c) The Raman spectrum of single layer MoSe2 on supported region. The A1g and E12g peaks are observed around ~240.6 and 286.4 cm-1 which is also sign of single layer MoSe2. Figure S3. (a) Schematic drawing of graphene FETs with Co and NiFe doping. (b) The resistivity vs back gate, Dirac measurements. (c) The RA of the junction devices before and after annealing. The resistance of all devices is reduced after annealing. Figure S4. The MR (%) values at different temperature for all type of devices after annealing by keeping current I = 10 μA. [file 11671_2020_3365_MOESM1_ESM.docx]

**Supplementary Information**

**Modulation of magnetoresistance polarity in BLG/SL-MoSe_2_ heterostacks**

Muhammad Farooq Khan^1^, Shania Rehman^1^, Malik Abdul Rehman^2^, Muhammad Abdul Basit^3^, Deok-kee Kim^1^, Faisal Ahmed^4,5^, H.M. Waseem Khalil^6^, Imtisal Akhtar^7^, and Seong Chan Jun^2^*

^1^Department of Electrical Engineering, Sejong University, 209 Neungdong-ro, Gwangjin-gu, Seoul 05006 Korea. ^2^School of Mechanical Engineering, Yonsei University, 50 Yonsei-ro, Seodaemun-gu, Seoul 03722, South Korea. ^3^Department of Materials Science and Engineering, Institute of Space Technology, Islamabad 44000, Pakistan ^4^Department of Mechanical Engineering, NUST College of Electrical and Mechanical Engineering, National University of Science and Technology, Islamabad, 44000, Pakistan. ^5^Department of Electronics and Nanoengineering, Aalto University, P.O. Box 13500, F1-00076 Aalto, Finland. ^6^Department of Electrical Engineering, College of Engineering and Technology, University of Sargodha, Pakistan. ^7^Department of Mechanical Engineering, Chung-Ang University, Seoul, South Korea.

**Corresponding Author**

*E-mail: [scj@yonsei.ac.kr](mailto:scj@yonsei.ac.kr)

**Supplementary Note 1. Fabrication of hole through wafers**

All devices were made using few-steps fabrication process as shown schematically in Supplementary Note 4. To make hole across the wafers, (SiN-300 nm/SiO_2_-300 nm/Si-600 μm/SiO_2_-300 nm/SiN-300 nm), we followed some steps with sequence. First of all, in 1^st^ step the LOR and photoresist (S1813) were spin-coated from the back side of the wafer to make 40 x 40 μm square by using photolithography. The SiN-300 nm was removed from this square area by inductively coupled plasma (ICP) dry etching system with CH_3_F_4_ gas plasma and following SiO_2_-300 nm was etched by using buffer oxide etchant (BOE) for 5 minutes. Subsequently, the Si-600 μm was etched by wet chemical method in which Si was exposed to KOH aqueous solution at 80 ^0^C until the top SiO_2_-300 nm is not revealed. Meanwhile after washing with distill water we dried the wafer by N_2_ blow carefully. Further in 2^nd^ step the upper SiO_2_-300 nm layer was again removed by BOE to render top thick free-standing SiN- 300 nm window. Finally, we spin coated the ZEP polymer on top of wafer for electron beam lithography to drill hole through suspended SiN membrane, thereby after e.beam lithography we expose circular hole area (~1 μm radius) by developing and etched by using again CH_3_F_4_ gas plasma. Later on, we wash and clean the wafer in hot PG remover and isopropyl alcohol (IPA). To ensure good adhesion between 2D material and SiN we did oxygen plasma by ICP to attain cleaner SiN surface particularly around hole. Eventually, the thick h-BN flake (necessary for only single layer graphene adhesion, otherwise SiN is not best option) was transferred on top of SiN hole by PMMA membrane and annealed on hot plate at 200 ^0^C for 2 min. Meanwhile, the reactive ion etching (RIE) was done from the back side of the wafer with CF_4_ plasma to etch h-BN completely from the suspended part. Simultaneously, the SiN membrane etched from the back side become thinner upto ~150 nm which is enough to suspend 2D materials and washed our devices in acetone to remove PMMA. Ultimately in 3^rd^ step the single layer graphene, larger than hole size, was transferred onto hole through h-BN by PMMA membrane. At this critical point, to remove PMMA we put our devices vertically in hot acetone and IPA. By using this strategy our efficiency to attain stable suspended single layer graphene is 99.9 %. Further, we annealed the devices in mixture of N_2_ and H_2_ gasses environment at 350 ^0^C for 4 hours to get rid from polymer residue. Finally, the Co and NiFe are deposited at top and from bottom of the suspended graphene, respectively.

**Supplementary Note 2. Schematic illustration of device fabrication for hole**


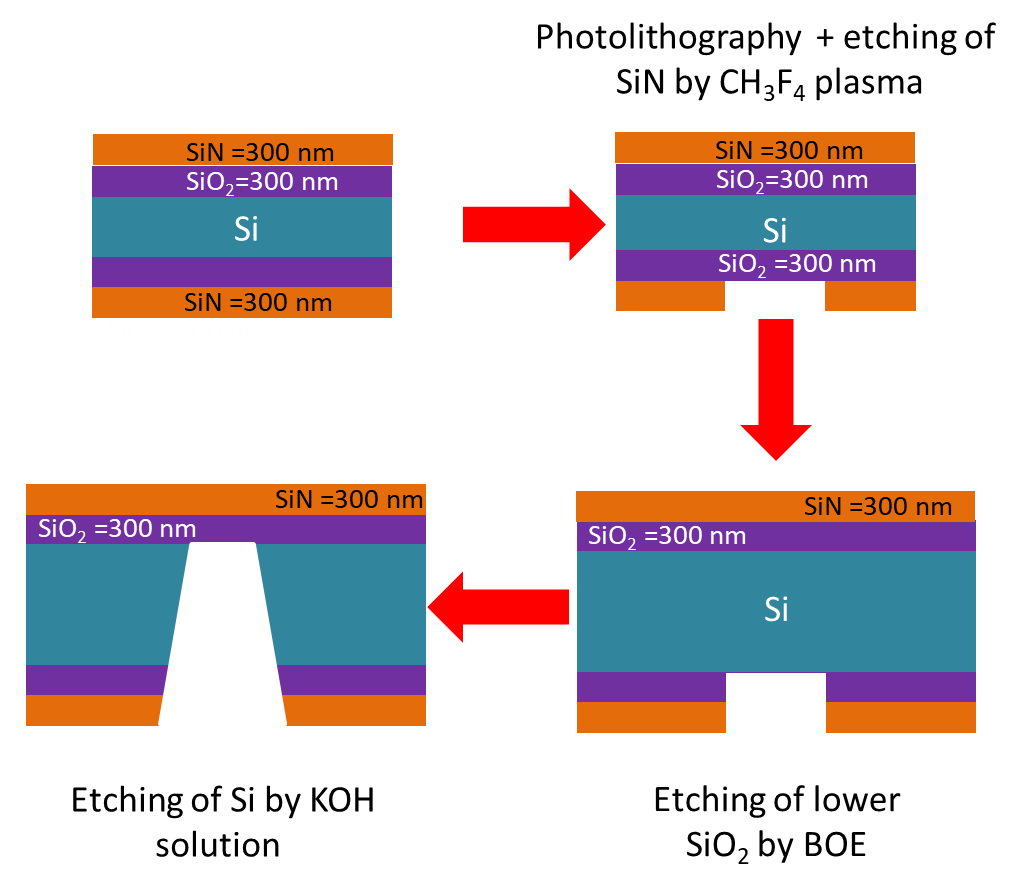


1^st^ step


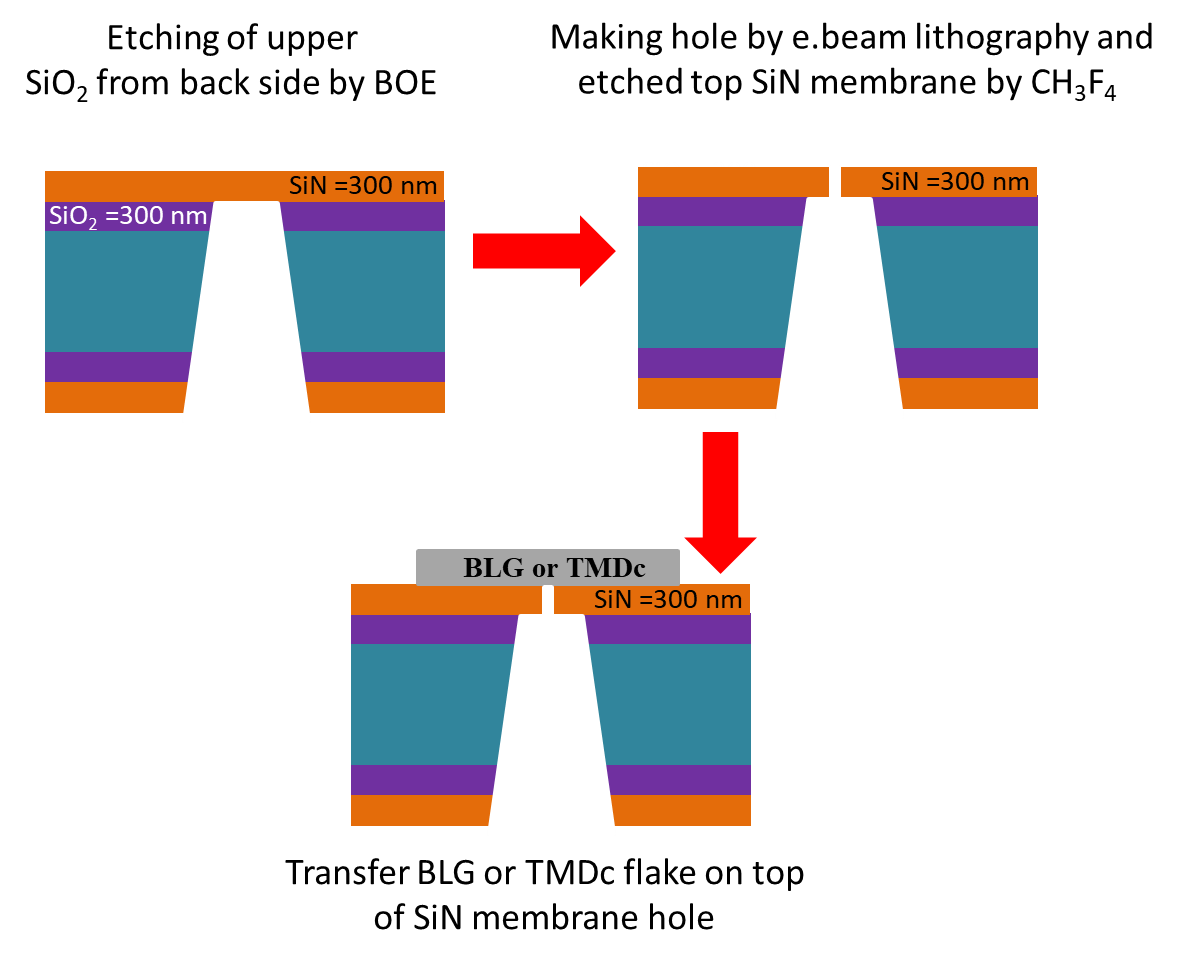


2^nd^ step


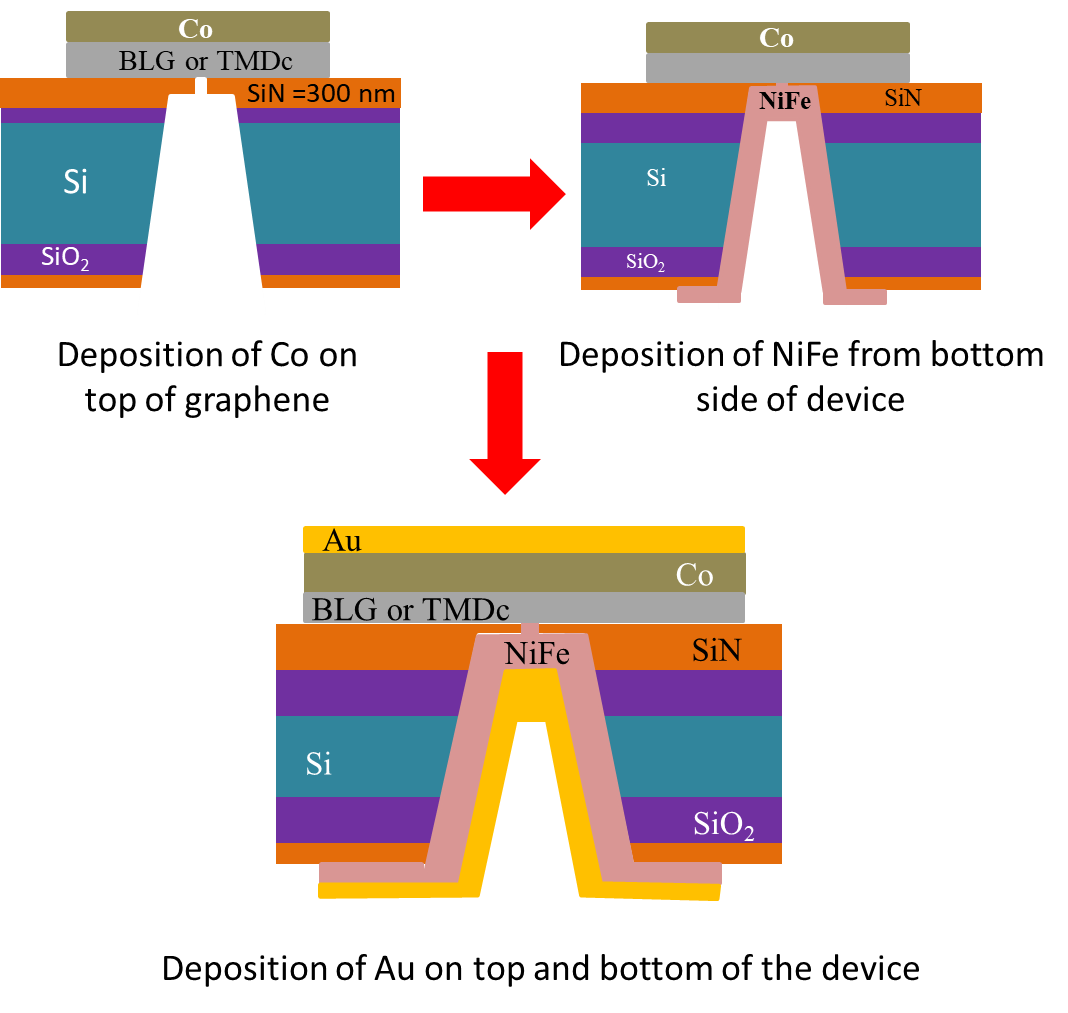


3^rd^ step


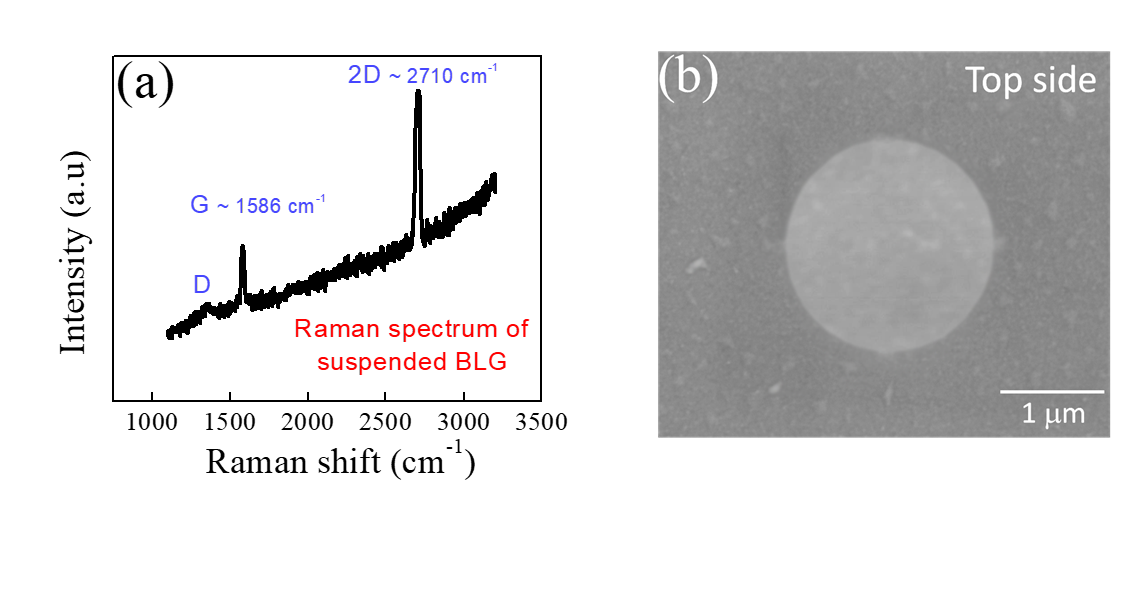


**Figure S1. Characterizations of suspended graphene structure**. **(a)** The Raman spectrum of bilayer suspended graphene. The small D peak is observed which attributed to strain effect and is normal in suspended graphene. **(b)** After FMs depositions the Scanning electron microscopy (SEM) image of final device from top side.


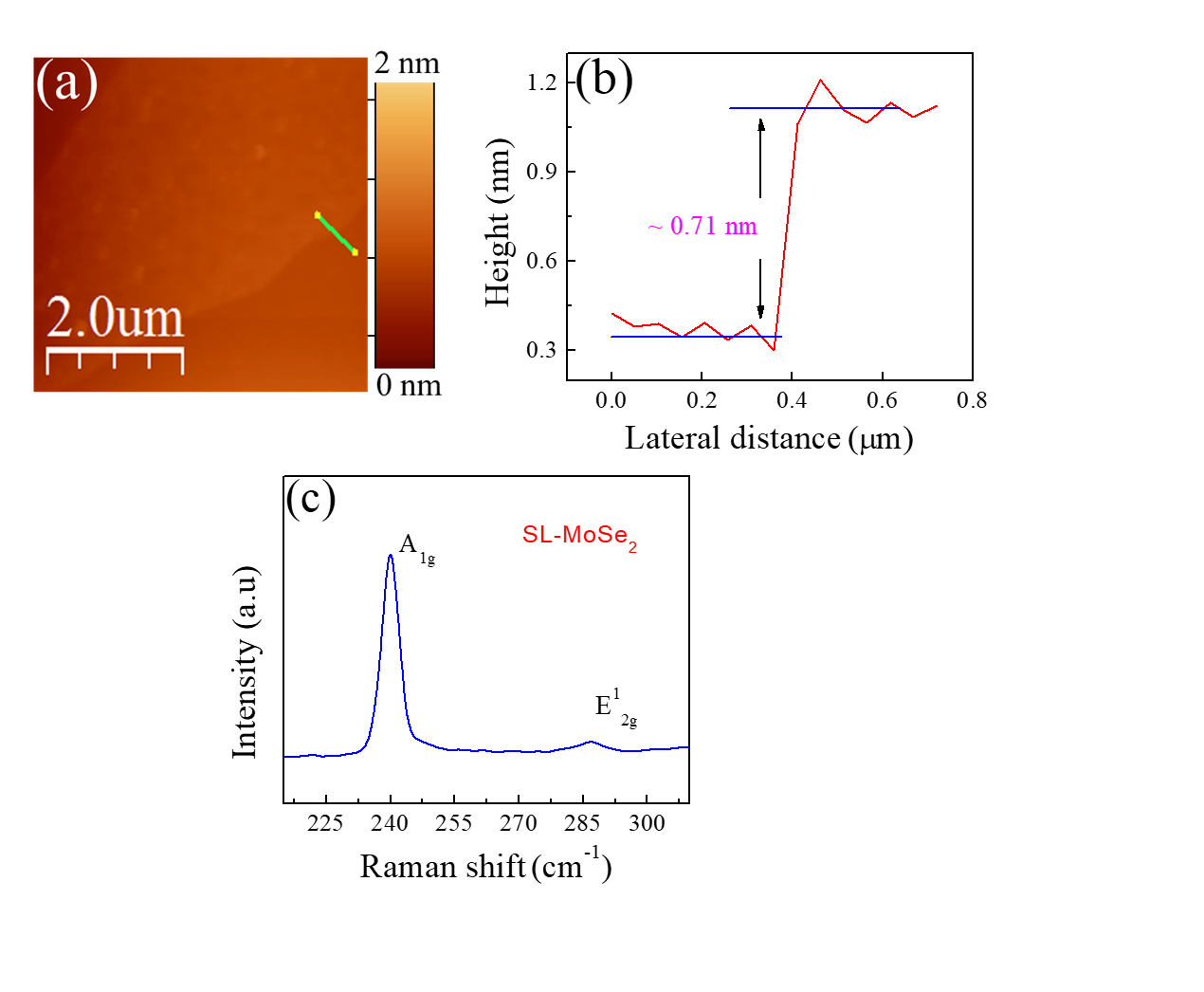


**Figure S2. (a)** The AFM image of single layer MoSe_2_ flake is taken on substrate. **(b)** The height profile corresponding to thin MoSe_2_ shows single layer as the thickness of our flake is very close to reported value (~0.77 nm). **(c)** The Raman spectrum of single layer MoSe_2_ on supported region. The A_1g_ and E^1^_2g_ peaks are observed around ~240.6 and 286.4 cm-1 which is also sign of single layer MoSe_2_.


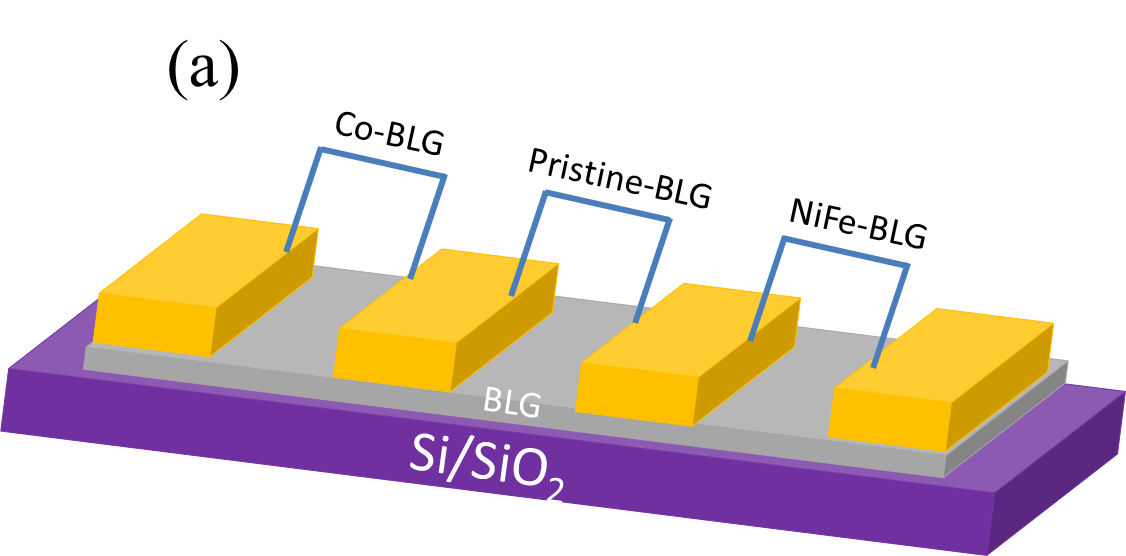

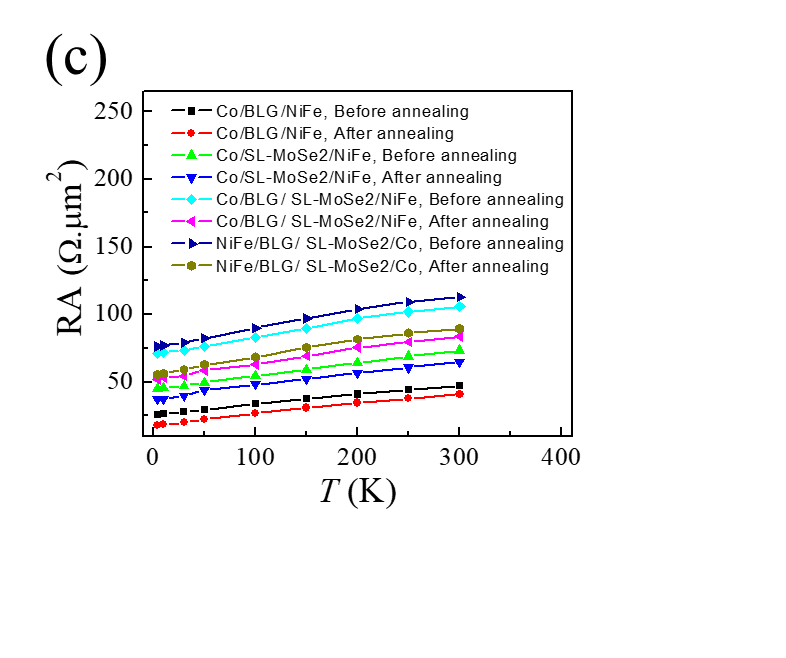


**Figure S3. (a)** Schematic drawing of graphene FETs with Co and NiFe doping. **(b)** The resistivity *vs* back gate, Dirac measurements. **(c)** The RA of the junction devices before and after annealing. The resistance of all devices is reduced after annealing.

**Figure S4**. The MR (%) values at different temperature for all type of devices after annealing by keeping current I = 10 μA.
